# Supplementary material for: Analysis of PPARγ Signaling Activity in Psoriasis
Source: Int J Mol Sci. 2021 Aug 10;22(16):8603. doi: 10.3390/ijms22168603 (PMC8395241; doi:10.3390/ijms22168603)
Supplement: Supplementary file 1 [file ijms-22-08603-s001.zip › Supplemental materials_Analysis of PPARg signaling activity in psoriasis/Pathway models/Models images and html files/PPARG inhibits phenotypes assotiated with psoriasis/143139.html]

innate immune response


# Cell Process innate immune response

|  |  |
| --- | --- |
| URN | urn:agi-gocellproc:0045087 |
| Total Entities | 1 |
| Connectivity | 4774 |
| Name | innate immune response |
| ObjectType | Cell Process |

---

|  |  |
| --- | --- |
| ChildConcepts | defense response signaling pathway |

---

|  |  |
| --- | --- |
| Pathway | SOCS in Negative Feedback Regulation in Myocarditis |
|  | Salivary Proteins Role in Periodontitis |
|  | PPARG inhibits phenotypes assotiated with psoriasis |
|  | Neighbors of innate immune response |

---

|  |  |
| --- | --- |
| MedScan ID | 4145087 |

---

|  |  |
| --- | --- |
| Alias | non specific immune responses |
|  | innate immune systems |
|  | natural immunity |
|  | nonspecific immune responses |
|  | innate immune responses |
|  | innate immune reactions |
|  | non specific immunities |
|  | innate immune reaction |
|  | nonspecific immune response |
|  | natural immunities |
|  | non specific immune response |
|  | innate immune system |
|  | nonspecific immunity |
|  | innate immunity |
|  | innate immunities |
|  | non specific immunity |
|  | innate immune response |
|  | nonspecific immunities |

---

|  |  |
| --- | --- |
| GO ID | 0045087 |

---
